# Supplementary material for: Population-level genome sequencing reveals distinct Mycobacterium tuberculosis intrahost mutational trajectories in simian immunodeficiency virus co-infected and antiretroviral treated non-human primates
Source: bioRxiv. 2026 Apr 4:2026.04.03.714442. Preprint. [Version 1] doi: 10.64898/2026.04.03.714442 (PMC13060361; doi:10.64898/2026.04.03.714442)

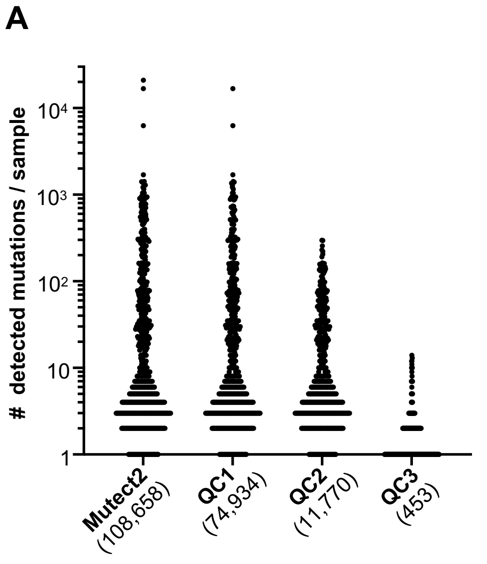

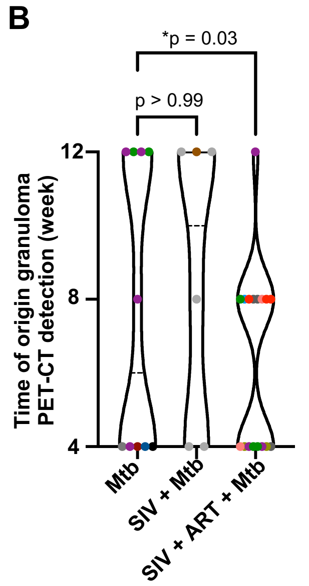

Supplementary Figure 1. (A) The number of genomic variants (parentheses) detected by Mutect2 per sequenced sample (dots) before and after quality control (QC) steps. (B) The time of PET-CT detection (either 4-, 8- or 12-weeks post infection) of a lung granuloma that served as an origin site for an in vivo mutation is compared across groups. Statistical test compared the distribution of 8 and 12-week lesions using a Kruskal-Wallis test with Dunn's correction. (C) STRING analyses showing the predicted interactions between all genes in the NHP dataset. Only interacting nodes are shown and are colored by their Mycobrowser category.

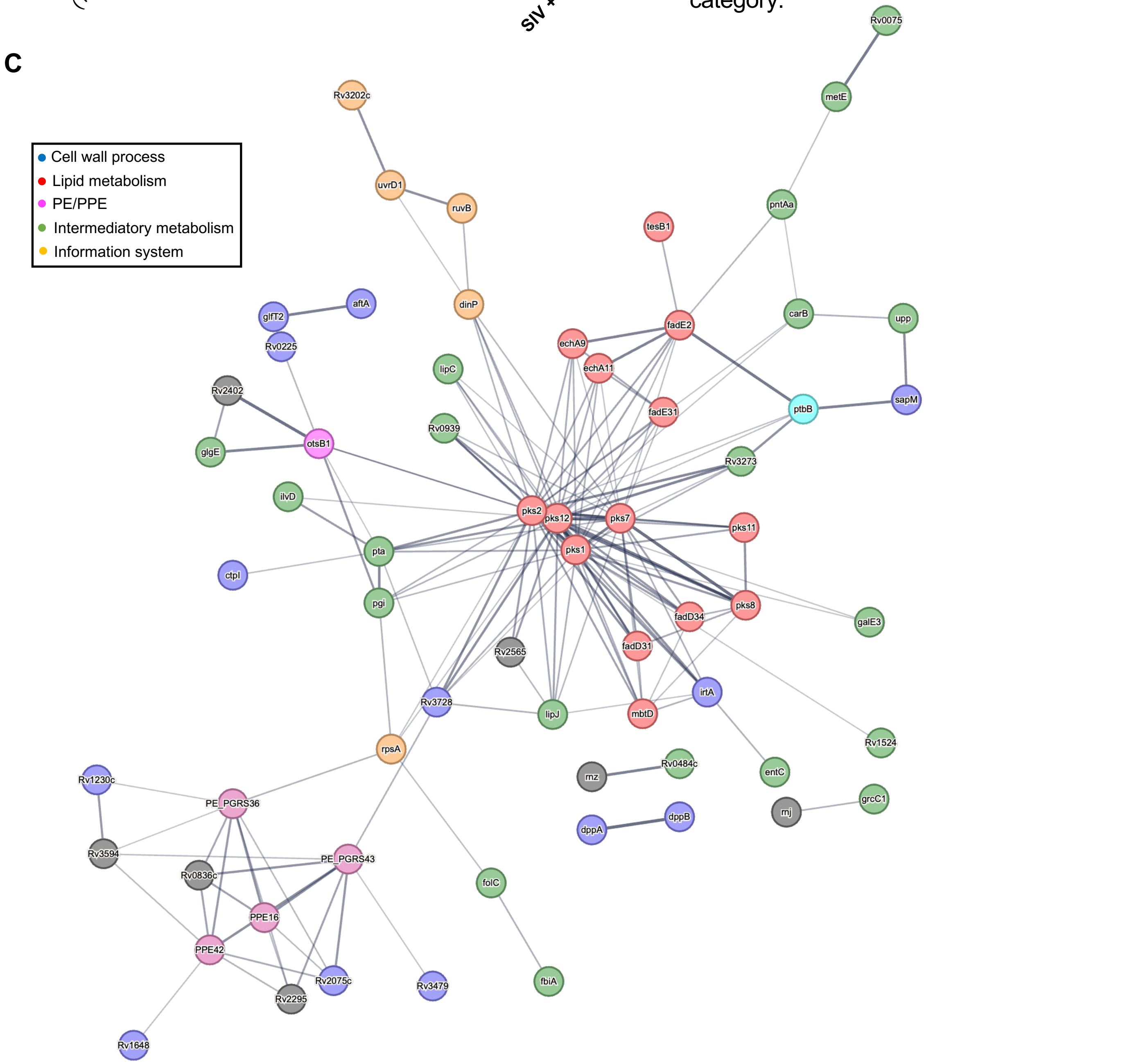

Supplement: Supplement 1 — Supplementary Figure 1. (A) The number of genomic variants (parentheses) detected by Mutect2 per sequenced sample (dots) before and after quality control (QC) steps. (B) The time of PET-CT detection (either 4-, 8- or 12-weeks post infection) of a lung granuloma that served as an origin site for an in vivo mutation is compared across groups. Statistical test compared the distribution of 8 and 12-week lesions using a Kruskal-Wallis test with Dunn’s correction. (C) STRING analyses showing the predicted interactions between all genes in the NHP dataset. Only interacting nodes are shown and are colored by their Mycobrowser category. [file media-1.pdf]
