# Supplementary material for: Population-level genome sequencing reveals distinct Mycobacterium tuberculosis intrahost mutational trajectories in simian immunodeficiency virus co-infected and antiretroviral treated non-human primates
Source: bioRxiv. 2026 Apr 4:2026.04.03.714442. Preprint. [Version 1] doi: 10.64898/2026.04.03.714442 (PMC13060361; doi:10.64898/2026.04.03.714442)

NHPs (all mutations)

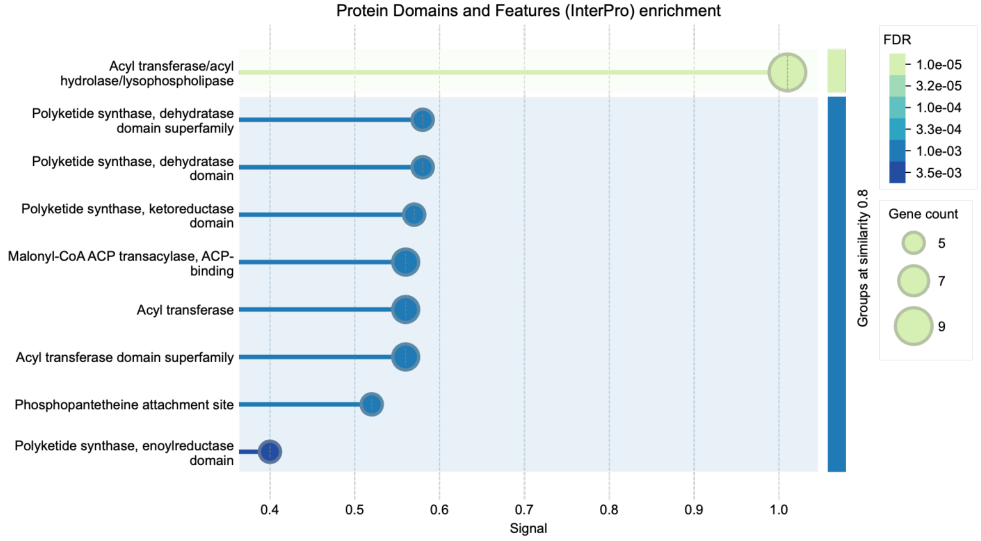

Supplementary Figure 2. All genes with intrahost mutations (all mutations = synonymous and non-synonymous mutations) for each dataset was analyzed for InterPro protein domain enrichment on the STRING database.

Liu et al. (all mutations)

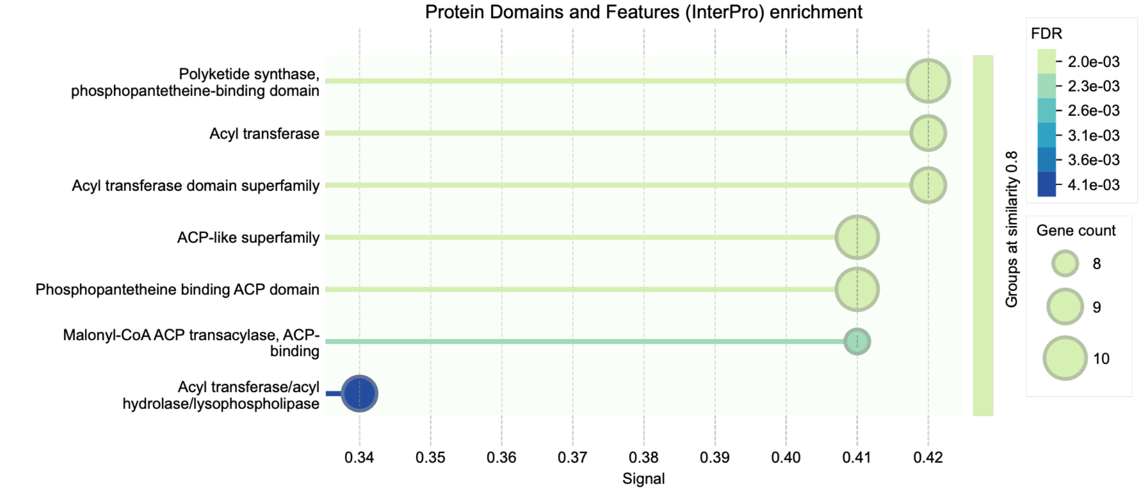

Lieberman et al. (non-synonymous mutations)

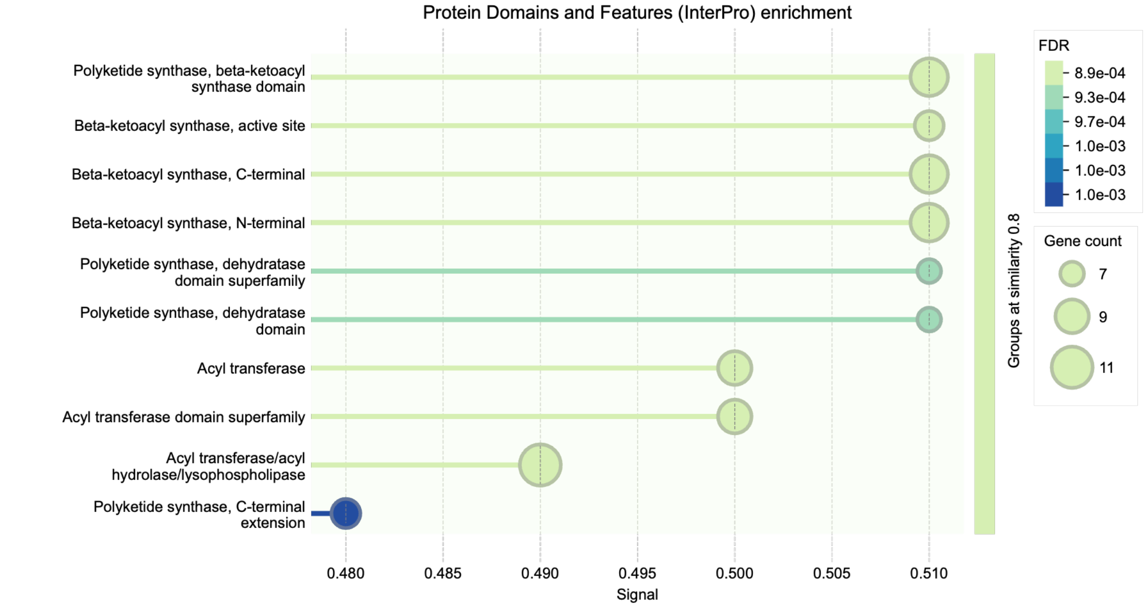

Supplement: Supplement 2 — Supplementary Figure 2. All genes with intrahost mutations (all mutations = synonymous and non-synonymous mutations) for each dataset was analyzed for InterPro protein domain enrichment on the STRING database. [file media-2.pdf]
